# Quality of Life of Patients with Heart Failure Due to Myocardial Ischemia

**DOI:** 10.31083/j.rcm2509342

**Published:** 2024-09-24

**Authors:** Grażyna Bonek-Wytrych, Oskar Sierka, Magdalena Szynal, Józefa Dąbek

**Affiliations:** ^1^College of Doctoral School, Faculty of Health Sciences in Katowice, Medical University of Silesia in Katowice, 40-752 Katowice, Poland; ^2^College of Doctoral School, Faculty of Medical Sciences in Katowice, Medical University of Silesia in Katowice, 40-752 Katowice, Poland; ^3^Department of Cardiology, Faculty of Health Sciences in Katowice, Medical University of Silesia in Katowice, 40-635 Katowice, Poland

**Keywords:** heart failure, quality of life, myocardial ischemia

## Abstract

**Background:**

Heart failure caused by myocardial ischemia is a common cause encountered in clinical practice. A significant problem in heart failure is its progressive, unfavorable course and the associated increased frequency of repeated hospitalizations, as well as a significant deterioration in the functioning of patients, leading to their inability to function independently and a significant decline in the quality of life. This study aimed to assess the quality of life of patients with heart failure due to myocardial ischemia, considering the left ventricular ejection fraction, the number of “diseased” coronary arteries, co-occurring diseases, and cardiovascular risk factors.

**Methods:**

A total of 204 patients with decompensated heart failure due to chronic myocardial ischemia were included in this study and examined twice: A: on the first day of hospitalization; A′: from 4 to 8 weeks. For the individual assessment of the quality of life, the WHOQOL-BREF (The World Health Organization Quality of Life - BREF) questionnaire was used.

**Results:**

In the group of patients with heart failure in the decompensation stage a statistically significant positive relationship was observed between the number of comorbidities and the social domain (R(A) = 0.197; *p*(A) = 0.005), the number of diseased coronary arteries and the mental (R(A) = 0.184; *p*(A) = 0.184) and environmental (R(A) = 0.149; *p*(A) = 0.034) domains, left ventricular ejection fraction (LVEF%) and quality of life (R(A) = 0.235; *p* = 0.001) and satisfaction with health (R(A) = 0.235; *p* = 0.001) and somatic domain (R(A) = 0.194; *p* = 0.005). A similar result was observed among patients in the long-term follow-up. A statistically significant negative correlation was demonstrated in the mentioned group between the LVEF% value and the social domain (R(A) = –0.235; *p* = 0.001), as well as in the long-term follow-up (R(A′) = –0.191, *p* = 0.026). The level of self-assessment of quality of life and satisfaction with health was statistically significantly higher among patients with heart failure in the long-term follow-up (3.20 ± 0.62) than in patients in the decompensation stage (1.98 ± 0.69).

**Conclusions:**

The quality of life of the examined patients with heart failure caused by chronic myocardial ischemia was poor, although it improved to average in the long-term follow-up. It was determined by left ventricular ejection fraction, the number of “diseased” coronary arteries, comorbidities, and risk factors.

## 1. Introduction

Cardiovascular diseases top the list of causes of death both worldwide and in Poland. They are responsible for up to 49% of all deaths in Europe and from 35 to 40% of deaths in Poland, even though their number is constantly decreasing [1, 2].

The number of patients with heart failure has doubled in the previous decades, which is why it is considered a social problem. The increased number of patients may be related to the observed population aging but also to the use of more and more effective methods for treating cardiovascular system diseases and, consequently, longer patient survivals [3].

According to the latest guidelines of the European Society of Cardiology (ESC) and the Polish Society of Cardiology (PTK), heart failure is a clinical syndrome consisting of subjective symptoms (shortness of breath, fatigue) accompanied by objective symptoms (increased pressure in the pulmonary veins, crackles pulmonary fields, peripheral edema) resulting from disturbances in the structure and/or function of the heart, leading to increased pressures in the heart chambers and/or inadequate cardiac output at rest or during exercise [4, 5, 6].

The most common causes of heart failure are coronary artery disease and hypertension. Secondly, excessive cardiac workload caused by, among others, valvular defects (aortic valve stenosis, mitral or tricuspid valve regurgitation, and aortic valve regurgitation) and cardiomyopathies (hypertrophic cardiomyopathy) should be indicated. Other causes include myocarditis, diabetes, constrictive pericarditis, and iatrogenic causes such as drugs and radiotherapy in mediastinal tumors, as well as oncological chemotherapy. A common reason is also toxic heart damage resulting from the abuse of alcohol or psychoactive substances [4, 5, 6].

Heart failure may develop due to diseases affecting the pericardium, myocardium, endocardium, and great vessels. It is most often associated with impaired left ventricular function. Systolic heart failure, regardless of its etiology, manifests itself in a decrease in cardiac output, which is related to many negative consequences. In the process of changes in the heart, the main role is played by the phenomenon of cardiac muscle reconstruction—remodeling [7]. The clinical study has revealed that in most patients after myocardial infarction, the intensive remodeling process is limited to the first weeks after the damage. However, it is sometimes progressive. Dilation leads to an increase in left ventricular wall stress and, ultimately, to the development of heart failure [7].

Heart failure caused by myocardial ischemia is one of the common causes encountered in clinical practice [8]. A significant problem in heart failure is its progressive, unfavorable course and the associated increased frequency of repeated hospitalizations, as well as a significant deterioration in the functioning of patients, leading to their inability to function independently with a significant decline in the quality of life.

According to the World Health Organization (WHO), quality of life is “an individual’s perception of his or her position in life in the cultural context and value system in which he or she lives and concerning the tasks, expectations, and standards determined by environmental conditions”. Quality of life indicators include the ability to play current life roles, adapt, and maintain psychological well-being and functioning within social groups [9]. Examining the quality of life in the medical concept means identifying problems resulting from the disease and the treatment used, relating to physical, mental, and social human activity, and describing the patient’s views on health and subjective well-being. In medicine, the quality of life is determined by health status (health-related quality of life—HRQoL) [10, 11].

## 2. Aim of the Study

This study aimed to assess the quality of life of patients with heart failure due to myocardial ischemia, considering the left ventricular ejection fraction, the number of “diseased” coronary arteries co-occurring diseases, and cardiovascular risk factors.

## 3. Materials and Methods

The research began after obtaining the consent of the Bioethics Committee of the Medical University of Silesia in Katowice no. PCN/0022/KB1/36/21 with supplement no. BNW/NWN/052/KB1/36/I/21/23.

The study group included patients with decompensated heart failure due to chronic myocardial ischemia after meeting the inclusion criteria: Aged over 18, consent to participate in the study, documented coronary artery disease (significantly narrowing lesions in the heart), coronary angiography, condition after coronary angioplasty or coronary artery bypass surgery and contractility disorders in transthoracic echocardiography, diagnosed heart failure in New York Heart Association (NYHA) functional classes III–IV. The exclusion criteria were lack of patient’s consent to participate in the study, lack of coronary heart disease confirmed through objective methods, heart failure based on other causes, shortness of breath caused by diseases in different organs (lungs, liver, kidneys), cancer, difficult contact (e.g., after a stroke), mental disorders.

A total of 204 patients with heart failure due to chronic ischemia (NYHA classes III/IV) were included in the study. A total of 156 (76.4%) men and 48 (23.5%) women were included in the study (204; 100%). The analyzed patients were 52 to 90 years old; the mean age was 69.8 ± 8.7 years. Since the examined patients were a group of seriously ill people, 31 people died during follow-up, while 37 did not report for long-term follow-up for an unknown reason (lack of contact).

Qualified patients were examined twice: A — on the first day of hospitalization; A′ — from 4 to 8 weeks. For the individual assessment of the quality of life and health status, the WHOQOL-BREF (The World Health Organization Quality of Life - BREF) quality of life questionnaire was used to assess the quality of life and four areas of life: physical, mental, social, and environmental, as well as questions subject to separate analysis: Regarding the individual general perception of the quality of life and the individual general perception of one’s health. The work involved transferring raw scores obtained in personal questions and domains in accordance with the questionnaire analysis protocol onto a point scale from 0 to 100, and the results were analyzed assuming point ranges [12, 13]: 0–20: very poor; 21–40: bad; 41–60: neither good nor bad; 61–80: good; 81–100: very good.

The chi-square test of independence was used to compare the feature frequency in groups/subgroups, Yates’ correction was applied if necessary, or Fisher’s exact test for small numbers was used (2 × 2 tables). Values of continuous variables were presented as the mean and standard deviation, minimum, and maximum values. The Mann–Whitney U test was used to measure data deviating from a normal distribution. Spearman’s rank correlation coefficient (R) was used in the correlation analysis, and its interpretation was made considering the ranges of values given by Schober P. *et al*. [14]. Statistical significance was assumed at <5% (*p* < 0.05). Statistical analysis was performed using the Statistica 13.0 PL program (StatSoft Polska, Kraków, Poland).

## 4. Results

Table 1 presents the general characteristics of the study group.

**Table 1.** **General characteristics of the studied group of patients with heart failure**.

Study group of patients with heart failure (n = 204; 100%)						
Variables					n	%
Sex				Woman	48	23.53
				Men	156	76.47
Age (years)				52–60	38	18.63
				61–70	56	27.45
M	SD	Min.	Max.	71–80	86	42.16
69.80	8.73	52	90	>80	24	11.76
Level of education				Primary	115	56.37
				Secondary	63	30.88
				Higher	26	12.75
Place of residence				City	169	82.84
				Village	35	17.16
Marital status				Miss/Bachelor	27	13.24
				Married	123	60.29
				Widow/Widower	54	26.47

**Abbreviations**: n, number of participants; M, average; SD, standard deviation; Min., minimum value; Max., maximum value.

The average age of the examined patients was 69.8 ± 8.73. The largest percentage were respondents with primary education (115; 56.37%), and in terms of place of residence — respondents from cities (169; 82.84%). Over 60% (123; 60.29%) of the respondents declared they were officially married.

Table 2 shows the characteristics of the study group, considering the occurrence of risk factors for cardiovascular diseases.

**Table 2.** **Characteristics of the studied group of patients with heart failure, considering the occurrence of classic risk factors for cardiovascular diseases**.

Study group of patients with heart failure (n = 204; 100%)					
Variables		A (n = 204; 100%)		A′ (n = 136; 100%)	
		n	%	n	%
Risk factors for cardiovascular diseases (question with multiple answer option)	Arterial hypertension	113	55.39	70	51.47
	Abdominal obesity	110	53.92	71	52.21
	Alcohol consumption	109	50.43	45	33.09
	Lack of physical activity/occasional activity	103	50.49	60	44.12
	Psychosocial factors	75	36.76	41	30.15
	Diabetes	72	35.29	54	39.71
	Hypercholesterolemia	70	34.31	28	20.59
	Smoking tobacco	68	33.33	20	14.71
	Improper nutrition	39	19.12	27	19.85
Number of risk factors present	0	1	0.49	2	1.47
	1	12	5.88	5	3.68
	2	32	15.69	20	14.71
	3	33	16.18	32	23.53
	4	32	15.69	23	16.91
	5	37	18.14	29	21.32
	6	32	15.69	13	9.56
	7	13	6.37	8	5.88
	8	7	3.43	4	2.94
	9	5	2.45	0	0.00
Test chi^2^: chi^2^ = 10.285, df = 9, *p* = 0.32					

**Abbreviations**: A, patients with heart failure in the decompensation stage; A′, patients with heart failure in long-term follow-up; n, number of participants; chi^2^, the value of the chi^2^ statistic; df, degrees of freedom; *p*, test probability.

The most frequently declared risk factor by patients with heart failure in the decompensation stage was hypertension (113; 55.39%), while in the long-term follow-up, it was abdominal obesity (71; 52.21%).

Table 3 presents the characteristics of the study group of patients with heart failure, including the occurrence of comorbidities.

**Table 3.** **Characteristics of the study group, including the occurrence of comorbidities**.

Study group of patients with heart failure (n = 204; 100%)			
Variables		A (n = 204; 100%)	
		n	%
Concomitant diseases	Coronary artery disease	204	100.00
	Heart attack	148	72.55
	Hypertension	113	55.39
	Diabetes	72	35.29
	Previous stroke	22	10.78
Number of comorbidities	1	6	2.94
	2	46	22.55
	3	71	34.80
	4	73	35.78
	5	8	3.92

**Abbreviations**: n, number; A, patients with heart failure in the decompensation stage.

All examined patients had coronary heart disease (204; 100.00%), and 73 (35.78%) declared the presence of four comorbidities.

**Fig. 1.**
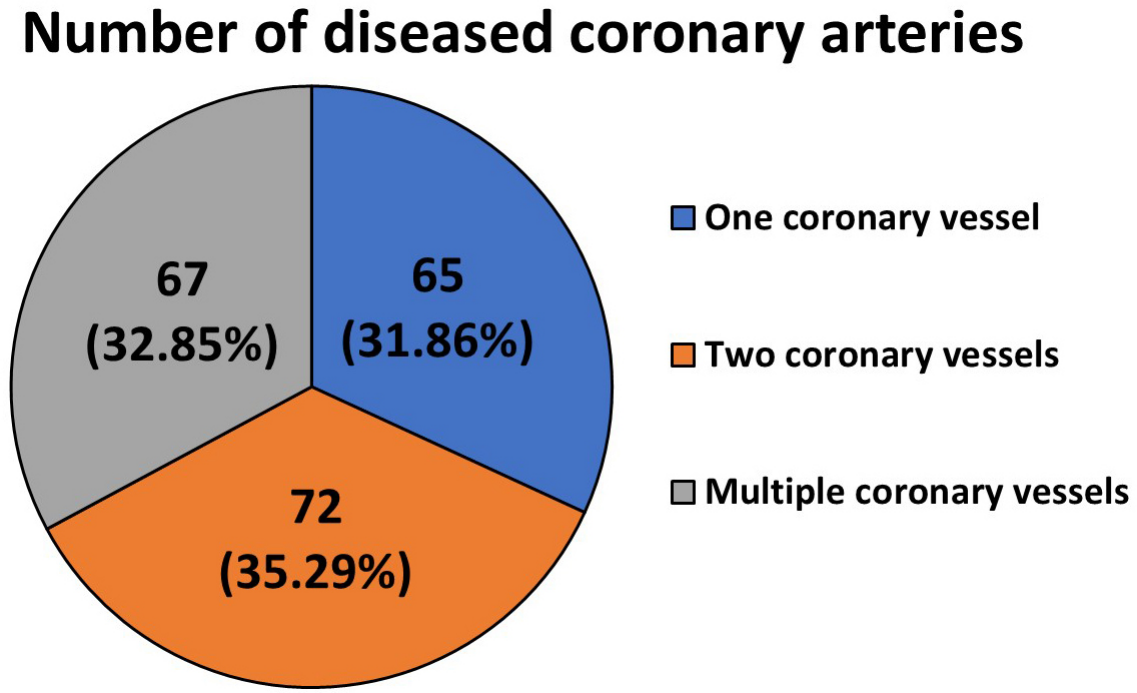
**Characteristics of the study group of patients with heart failure, including the number of diseased coronary arteries**.

The characteristics of the study group of patients with heart failure, considering the number of diseased coronary arteries, are presented in Fig. 1.

Most respondents were patients with atherosclerotic lesions detected in two coronary vessels (72; 35.29%).

Table 4 presents the characteristics of the study group of patients with heart failure, including the values of left ventricular ejection fraction (LVEF) and the number of people in particular phenotypic classes of heart failure.

**Table 4.** **Characteristics of the studied group of patients with heart failure, considering the value of left ventricular ejection fraction (LVEF) and the number of people in particular phenotypic classes of heart failure**.

Study group of patients with heart failure (n = 204; 100%)								
LVEF	A (n = 204; 100%)				A′ (n = 136; 100%)			
	M	SD	Min.	Max.	M	SD	Min.	Max.
	30.16	10.68	14.00	53.00	36.77	9.796	21.00	61.00
Mann–Whitney U test (with continuity correction)	Z = –5.771, *p * < 0.001*							
Heart failure phenotype based on LVEF	A (n = 204; 100%)				A′ (n = 136; 100%)			
	n		%		n		%	
Heart failure with reduced ejection fraction (HFrEF) (LVEF: ≤40%)	166		81.37		86		63.24	
Heart failure with mildly reduced ejection fraction (HFmrEF) (LVEF: 41–49%)	17		8.33		32		23.53	
Heart failure with preserved ejection fraction (HFpEF) (LVEF: ≥50%)	21		10.29		18		13.24	

**Abbreviations**: n, number of participants; M, average; SD, standard deviation; Min., minimum value; Max., maximum value; A, patients with heart failure in the decompensation stage; A′, patients with heart failure in long-term follow-up; Z, Mann–Whitney U test value; *p*, test probability; *, statistically significant result.

The Mann–Whitney U test showed a statistically significant difference (*p* < 0.0001) between the LVEF values in patients in the decompensation stage and long-term follow-up. This means that patients with heart failure in the decompensation stage had significantly lower ejection fraction values than in the long-term follow-up.

The study group’s characteristics, considering the relationship between the number of comorbidities and the quality of life, are presented in Table 5.

**Table 5.** **Characteristics of the study group of patients with heart failure, considering the correlation between the number of comorbidities and the quality of life**.

Study group of patients with heart failure (n = 204; 100%)		
Variable	A (n = 204; 100%)	
	R(A)	*p*(A)
Number of comorbidities and quality of life	0.030	0.670
Number of comorbidities and satisfaction with health condition	0.030	0.670
Number of comorbidities and somatic domain	0.069	0.324
Number of comorbidities and psychic domain	–0.003	0.969
Number of comorbidities and social domain	0.197	0.005*
Number of comorbidities and environmental domain	0.019	0.791

**Abbreviations**: n, number of participants; R, Spearman’s rank correlation coefficient value; *p*, test probability; A, patients with heart failure in the decompensation stage; *, statistically significant result.

The Spearman’s rank correlation test showed a weak, statistically significant positive relationship between the number of comorbidities and the social domain (R(A) = 0.197; *p*(A) = 0.005) in the group of patients with heart failure in the decompensation stage. This means that patients with more comorbidities assessed activities in the social domain better.

Table 6 presents the characteristics of the study group of patients with heart failure, including the relationship between the left ventricular ejection fraction (LVEF%) and the quality of life.

**Table 6.** **Characteristics of the study group of patients with heart failure, including the relationship between the left ventricular ejection fraction (LVEF%) and the quality of life**.

Study group of patients with heart failure (n = 204; 100%)				
Variable	A (n = 204; 100%)		A′ (n = 136; 100%)	
	R(A)	*p*(A)	R(A′)	*p*(A′)
LVEF% and quality of life	0.235	0.001*	0.383	<0.001*
LVEF% and satisfaction with health condition	0.235	0.001*	0.383	<0.001*
LVEF% and somatic domain	0.194	0.005*	0.142	0.098
LVEF% and psychic domain	–0.009	0.902	–0.001	0.995
LVEF% and social domain	–0.235	0.001*	–0.191	0.026*
LVEF% and environmental domain	–0.107	0.128	–0.010	0.911

**Abbreviations**: n, number of participants; A, patients with heart failure in the decompensation stage; A′, patients with heart failure in long-term follow-up; R, Spearman’s rank correlation coefficient value; *p*, test probability; *, statistically significant result.

The Spearman’s rank correlation test showed weak, statistically significant positive relationships between LVEF% and quality of life (R(A) = 0.235; *p* = 0.001) and satisfaction with health (R(A) = 0.235; *p* =0.001), as well as the somatic domain (R(A) = 0.194; *p* = 0.005) in the group of patients with heart failure in the decompensating stage, which means that people with a higher left ventricular ejection fraction assessed the above-mentioned issues better. These results were similar among patients in the long-term follow-up: a positive relationship between the LVEF% value and the quality of life (R(A′) = 0.383, *p* < 0.001) and satisfaction with health (R(A′) = 0.383, *p* < 0.001).

However, a statistically significant negative correlation was demonstrated in the group of patients with heart failure in the decompensation stage between the LVEF% value and the social domain (R(A) = –0.235; *p* = 0.001) and also in the long-term follow-up and the social domain (R(A′) = –0.191, *p = *0.026*; *, statistically significant result.), which means that patients with a higher left ventricular ejection fraction rated the activities in the mentioned domain worse.

Table 7 presents the characteristics of the study group of patients with heart failure, considering the correlation between the number of diseased coronary arteries and the quality of life.

**Table 7.** **Characteristics of the study group of patients with heart failure, considering the correlation between the number of diseased coronary arteries and the quality of life**.

Study group of patients with heart failure (n = 204; 100%)		
Variable	A (n = 204; 100%)	
	R(A)	*p*(A)
Number of diseased coronary arteries and quality of life	–0.009	0.897
Number of diseased coronary arteries and satisfaction with health condition	–0.009	0.897
Number of diseased coronary arteries and somatic domain	0.071	0.313
Number of diseased coronary arteries and psychic domain	0.184	0.008*
Number of diseased coronary arteries and social domain	0.119	0.090
Number of diseased coronary arteries and environmental domain	0.149	0.034*

**Abbreviations**: n, number of participants; A, patients with heart failure in the decompensation stage; R, Spearman’s rank correlation coefficient value; *p*, test probability; *, statistically significant result.

In the group of patients with heart failure in the decompensation stage, Spearman’s rank correlation test showed weak, statistically significant positive correlations between the number of diseased coronary arteries and the mental (R(A) = 0.184; *p*(A) = 0.184) and environmental domain (R(A) = 0.149; *p*(A) = 0.034). This means that the examined patients with a larger number of coronary arteries assessed their activities better in the mental and environmental domains.

Table 8 presents the characteristics of the study group of patients with heart failure, considering the correlation between the number of risk factors and the quality of life.

**Table 8.** **Characteristics of the studied group of patients with heart failure, considering the correlation between the number of risk factors and the quality of life**.

Study group of patients with heart failure (n = 204; 100%)				
Variable	A (n = 204; 100%)		A′ (n = 136; 100%)	
	R(A)	*p*(A)	R(A′)	*p*(A′)
Number of risk factors present and quality of life	–0.076	0.281	–0.1428	0.097
Number of risk factors present and satisfaction with health condition	–0.076	0.281	–0.1428	0.097
Number of risk factors present and somatic domain	–0.122	0.083	–0.1068	0.216
Number of risk factors present and psychic domain	–0.138	0.049*	–0.212	0.013*
Number of risk factors present and social domain	0.001	0.983	0.180	0.036*
Number of risk factors present and environmental domain	–0.215	0.002*	–0.0629	0.467

**Abbreviations**: n, number of participants; A, patients with heart failure in the decompensation stage; A′, patients with heart failure in long-term follow-up; R, Spearman’s rank correlation coefficient value; *p*, test probability; *, statistically significant result.

In the group of patients with heart failure in the decompensation stage, Spearman’s rank correlation test showed weak, statistically significant negative correlations between the number of risk factors and the mental domain (R(A) = –0.138; *p* = 0.049) and the environmental domain (R(A) = –0.215; *p* = 0.002), while in the long-term follow-up there was a negative correlation between the number of risk factors and the mental domain (R(A) = –0.212; *p* = 0.013) and a positive correlation with the social domain (R(A) = 0.180; *p* = 0.036).

**Supplementary Table 1** presents the characteristics of the study group of patients in the heart failure decompensation stage and the follow-up, considering their quality of life and the presence of classic risk factors. Table 9 presents the analysis of factors contributing to reduced quality of life in patients with heart failure in the decompensation stage.

**Table 9.** **Assessment of factors contributing to reduced quality of life in patients with heart failure in the decompensation stage**.

Study group of patients with heart failure in decompensation stage (n = 204; 100%)				
Dependent variable	Factor—predictor	Predictor value	OR (95% CI)	*p*
Reduced quality of life	Psychosocial factors	No (n = 129)	2.52 (1.17–5.43)	0.019
		Yes (n = 75)		

**Abbreviations**: n, number of participants; OR, odds ratio; CI, confidence interval; *p*, test probability.

A statistically significant relationship was demonstrated only between the occurrence of psychosocial factors and the quality of life among patients in the decompensation stage. The only risk factor for reduced quality of life among them was psychosocial factors, increasing the risk of reduced quality of life by approximately 2.5 times.

Table 10 presents the characteristics of the study group, including an assessment of their quality of life.

**Table 10.** **Characteristics of the study group of heart failure patients, including the quality of life assessment results**.

Study group of patients with heart failure (n = 204; 100%)								
Variable		M	SD	Med.	Min.	Max.	Mann–Whitney U test	
							Z	*p*
Quality of life	A	1.98	0.69	2.00	1.00	3.00	–12.321	<0.001*
(1–5)	A′	3.20	0.62	3.00	2.0	4.00		
Satisfaction with health condition	A	1.98	0.69	2.00	1.00	3.00	–12.321	<0.001*
(1–5)	A′	3.20	0.62	3.00	2.00	4.00		
Somatic domain	A	43.16	9.35	44.00	19.00	63.00	–12.101	<0.001*
(0–100)	A′	62.96	13.93	56.00	38.00	94.00		
Psychic domain	A	41.61	7.40	44.00	25.00	56.00	–13.833	<0.001*
(0–100)	A′	58.45	7.87	56.00	44.00	75.00		
Social domain	A	50.54	11.51	50.00	25.00	75.00	–14.195	<0.001*
(0–100)	A′	75.63	11.93	75.00	50.00	100.00		
Environmental domain	A	49.71	10.57	50.00	19.00	75.00	–14.162	<0.001*
(0–100)	A′	75.20	11.03	75.00	50.00	100.00		

**Abbreviations**: n, number of participants; M, mean; SD, standard deviation; Med., median; Min., minimum value; Max., maximum value; A, patients with heart failure in the decompensation stage; A′, patients with heart failure in long-term follow-up; Z, Mann–Whitney U test value; *p*, test probability; *, statistically significant result.

The Mann–Whitney U test showed that the level of self-assessment of quality of life and satisfaction with health was statistically significantly higher among patients with heart failure in the long-term follow-up (3.20 ± 0.62) than in patients in the decompensation stage (1.98 ± 0.69). Moreover, it was shown that the respondents perceived their quality of life and satisfaction with their health similarly.

## 5. Discussion

All subjects had coronary artery disease (204; 100.00%), and 148 (72.55%) had suffered at least one heart attack. Most examined patients had atherosclerotic lesions detected in two coronary vessels (72; 35.29%). As confirmed by the literature, coronary artery disease and increasing myocardial ischemia resulting from the deposition of atherosclerotic plaques in the coronary arteries is one of the most common causes of heart failure [4, 5, 6]. In third place, the surveyed patients mentioned hypertension (113; 55.39%). Previous studies have indicated that arterial hypertension is one of the modifiable risk factors for cardiovascular diseases, including heart failure [15, 16, 17]. The surveyed patients with heart failure in the decompensation stage also answered that arterial hypertension (113; 55.39%) is the most common of the classic cardiovascular risk factors.

In the study group of patients with heart failure, the relationship between risk factors and the quality of life assessment using the WHOQOL-BREF questionnaire was also checked. A statistically significant relationship was found only among patients with heart failure in the decompensation stage between psychosocial factors and reduced quality of life. The risk factor for decreased quality of life was approximately 2.5 times. This is consistent with the previously mentioned research results on anxiety disorders and depression among patients with heart failure [18, 19, 20, 21, 22]. Psychosocial factors, such as stress and negative emotions, are associated with a lower quality of life, which was confirmed by the research of Żołnierczuk-Kieliszek D *et al*. [23], who showed that the worst subjective assessment of the quality of life in the group of cardiological patients was characterized by subjects suffering from chronic stress, and the occurrence of negative emotions was associated with pain (bodly pain (BP), *p* < 0.001) and their social functioning (SF, *p* = 0.001). In their review, Szyguła-Jurkiewicz *et al*. [24] compiled many studies in which stress significantly reduced the quality of life in patients with heart failure. In the study by N. Rod *et al*. [25], almost a quarter of the study population reported life exhaustion, which was associated with a higher risk of heart failure (*p* < 0.002). High life exhaustion was associated with an almost 2-fold higher risk of heart failure in men (hazard ratio = 1.93, 95% confidence interval: 1.20, 3.10) and a 2.5-fold higher risk in women (hazard ratio = 2.56, 95% confidence interval: 1.80, 3.65) [25]. However, in the study by Ogilvie RP *et al*. [26], there was no relationship between psychosocial factors and the risk factors for developing heart failure (95% confidence interval).

The WHOQOL-BREF questionnaire was used to assess the quality of life of patients in our study. This study showed that the level of self-assessment of quality of life and satisfaction with health was significantly higher among patients with heart failure in the long-term follow-up (3.20 ± 0.62) than in the decompensation stage (1.98 ± 0.69). Similar relationships were also demonstrated, considering the score results in individual domains assessed in the above-mentioned questionnaire. The individual scores were higher among patients in the long-term follow-up stage than those in the decompensation stage in each of the four domains: somatic, social, mental, and environmental.

In their study of patients with chronic heart failure, Wiśnicka P. *et al*. [27] showed an average self-assessment of quality of life of 2.78 points (SD = 0.91), which lower than the result in our study. In the cited studies, patients assessed their quality of life best in the environmental domain (average (M) = 13.28; SD = 3.11), followed by the social domain (M = 12.81; SD = 2.71), and the psychological domain (M = 12.8; SD = 3.2). However, the quality of life was rated the worst in the physical domain (M = 10.44; SD = 2.85) [27]. In our study, over half (108; 52.94%) of the respondents with heart failure in the decompensation stage assessed their quality of life as poor, and almost 30% (40; 29.41%) of patients in the long-term follow-up assessed their quality of life as good. However, less than ¼ (46; 22.55%) of patients with heart failure in the decompensation stage assessed their quality of life as “neither good nor bad”, and just over 10% (17; 12.50%) of patients assessed their quality of life in the long-term follow-up as poor. Alharbi M *et al*. [28] found that in their group of patients with heart failure tested using the SP-36 questionnaire, health-related quality of life was also moderate.

Kowalczyk B. *et al*. [29] examined heart failure patients using the Nottingham Health Profile (NHP) questionnaire to consider their quality of life. The analysis of the above-mentioned questionnaire showed that a higher quality of life determined better disease acceptance and was adversely affected by mobility limitations, social alienation, emotional reactions, energy level, cardiac ejection fraction level, and some comorbidities [29]. Our study showed that people with a higher level of cardiac ejection fraction provided a better assessment of their quality of life (R(A) = 0.235; *p* = 0.001) and satisfaction with health (R(A) = 0.235; *p* = 0.001), as well as the somatic (R(A) = 0.194; *p = *0.005). It was similar among patients in the long-term follow-up—a positive relationship between the LVEF% value and the quality of life (R(A′) = 0.383, *p* < 0.001) and satisfaction with health (R(A′) = 0.383, *p* < 0.001). The same conclusions were reached by AbuRuz M. *et al*. [30], who examined the quality of life among patients in Saudi Arabia. In the studies of the above-mentioned authors, a higher ejection fraction was also associated with a higher assessment of the quality of life by the patients [30]. The same relationship was also demonstrated in the study by Pelegrino VM *et al*. [31]. Our study also revealed that the size of the fraction positively correlates with the somatic domain of the WHOQOL-BREF test among patients with heart failure in the decompensation stage, which means that the higher the fraction possessed by the subjects, the better they assessed their health. However, a negative correlation was demonstrated with the social domain, both in the stage of decompensation and long-term follow-up (R(A) = –0.235, *p* = 0.001 vs. (R(B) = –0.191, *p* = 0.026*).

Our study showed that assessing the patient’s quality of life is closely related to their health condition and improves simultaneously. Determining the quality of life using health is a very important issue. Although it has interested researchers for many years, it is worth researching new groups of patients to seek new conclusions.

The study was not conducted free from limitations. The most important was the study group, which was difficult to assemble. The study group consisted of people who were seriously ill due to heart failure caused by chronic myocardial ischemia and were in the decompensation stage during the first part of the examination (functional classes III–IV, according to NYHA). For this reason, these patients often refused to participate in the study due to their health condition and poor well-being during hospitalization. Usually, people included in the study were no longer present during the second part of the study from 4 to 8 weeks in the long-term follow-up, probably due to poorer well-being or a reluctance to continue participating in the study; many of them did not appear for an unknown reason (lack of contact) or death.

This study showed that many seriously ill patients, often hospitalized due to exacerbations of heart failure, still possessed risk factors, such as arterial hypertension: A: 55.39% and A′: 51.47%; abdominal obesity: A: 53.92% and A′: 52.21%; diabetes: A: 35.29% and A′: 39.71%; improper nutrition: A: 19.12% and A′: 19.85%. In the long-term follow-up, as many as 44.12% of respondents declared little or no physical activity. This showed how poor the knowledge of the examined patients regarding health control and prevention of heart failure exacerbations was. The obtained results indicate the need to conduct systematic health education in this area to eliminate factors influencing the decompensation of the disease and improve the quality of life of patients in this group.

## 6. Conclusions

(1) The quality of life of the examined patients with heart failure caused by chronic myocardial ischemia in the decompensation stage was poor and determined by the left ventricular ejection fraction, as well as the number of “diseased” coronary arteries and risk factors; however, it improved to average in the long-term follow-up.

(2) The examined patients had numerous risk factors, in particular arterial hypertension, abdominal obesity, alcohol consumption, and low physical activity, while psychosocial factors increased the risk of its decline only in the decompensation stage when they were related to the quality of life.

(3) The number of comorbidities in the decompensation stage correlated positively with the social domain of the subjects’ quality of life. Furthermore, the more risk factors were present, the worse the patients assessed their quality of life in the mental and environmental domains (negative correlation). Moreover, in the long-term follow-up, the worse patients assessed their quality of life in the mental domain, the better the social domain.

## Availability of Data and Materials

The datasets used and/or analyzed during the current study are available from the corresponding author on reasonable request.

## Supplementary Material

Supplementary material associated with this article can be found, in the online version, at https://doi.org/10.31083/j.rcm2509342.
