# Supplementary material for: Quality of Life of Patients with Heart Failure Due to Myocardial Ischemia
Source: Rev Cardiovasc Med. 2024 Sep 24;25(9):342. doi: 10.31083/j.rcm2509342 (PMC11440393; doi:10.31083/j.rcm2509342)
Supplement: Supplementary file 1 [file 2153-8174-25-9-342-s1.docx]

**Supplementary Table 1. Assessment of the quality of life of patients with heart failure in the decompensation stage and long-term follow-up, taking into account the risk factors for
ischemic heart disease**

| **Variables** | | **Stage of decompensation (n=204; 100%)** | | | | | **Patients in follow-up (n=136; 100%)** | | | | | |
| --- | --- | --- | --- | --- | --- | --- | --- | --- | --- | --- | --- | --- |
|  |  | **Quality of life – reduced** | **Quality of life - Neutral or good** | **Chi^2^** | **df** | **p** | **Variables** | **Quality of life – reduced** | **Quality of life - Neutral or good** | **Chi^2^** | **df** | **p** |
| **Diabetes type 2** | Yes (72; 100%) | 51 (70.83%) | 21 (29.17%) | 2.79 | 1 | 0.09 | Yes (54; 100%) | 10 (18.52%) | 44 (81.48%) | 2.97 | 1 | 0,09 |
|  | No (132; 100%) | 107 (81.06%) | 25 (18.94%) |  |  |  | No (82; 100%) | 7 (8.54%) | 75 (91.46%) |  |  |  |
| **Arterial hypertension** | Yes (113; 100%) | 87 (76.99%) | 26 (23.01%) | 0.03 | 1 | 0.86 | Yes (70; 100%) | 9 (12.86%) | 61 (87.14%) | 0.017 | 1 | 0.89 |
|  | No (91; 100%) | 71 (78.02%) | 20 (21.98%) |  |  |  | No (66; 100%) | 8 (12.12%) | 58 (87.88%) |  |  |  |
| **Smoking tobacco** | Yes (68; 100%) | 56 (82.35%) | 12 (17.65%) | 1.40 | 1 | 0.24 | Yes (20; 100%) | 3 (15.00%) | 17 (85.00%) | 0.134 | 1 | 0.71 |
|  | No (136; 100%) | 102 (75.00%) | 34 (25.00%) |  |  |  | No (116; 100%) | 14 (12.07%) | 102 (87.93%) |  |  |  |
| **Alcohol consumption** | Yes (109; 100%) | 81 (74.31%) | 28 (25.69%) | 1.32 | 1 | 0.25 | Yes (45; 100%) | 3 (6.67%) | 42 (93.33%) | 2.09 | 1 | 0.15 |
|  | No (95; 100%) | 77 (81.05%) | 18 (18.95%) |  |  |  | No (91; 100%) | 14 (15.38%) | 77 (84.62%) |  |  |  |
| **Phycical activity** | Yes (101; 100%) | 73 (72.28%) | 28 (27.72%) | 3.07 | 1 | 0.08 | Yes (76; 100%) | 6 (7.89%) | 70 (92.11%) | 3.34 | 1 | 0.07 |
|  | No (103; 100%) | 85 (82.52%) | 18 (17.48%) |  |  |  | No (60; 100%) | 11 (18.33%) | 49 (81.67%) |  |  |  |
| **Hypercholesterolemia** | Yes (70; 100%) | 50 (71.43%) | 20 (28.57%) | 2.21 | 1 | 0.14 | Yes (28; 100%) | 5 (17.86%) | 23 (82.14%) | 0.93 | 1 | 0.34 |
|  | No (134; 100%) | 108 (80.60%) | 26 (19.40%) |  |  |  | No (108; 100%) | 12 (11.11%) | 96 (88.89%) |  |  |  |
| **Abdominal obesity** | Yes (110; 100%) | 84 (76.36%) | 26 (23.64%) | 0.16 | 1 | 0.69 | Yes (71; 100%) | 10 (14.08%) | 61 (85.92%) | 0.34 | 1 | 0.56 |
|  | No (94; 100%) | 74 (78.72%) | 20 (21.28%) |  |  |  | No (65; 100%) | 7 (10.77%) | 58 (89.23%) |  |  |  |
| **Improper nutrition** | Yes (38; 100%) | 30 (78.95%) | 8 (21.05%) | 0.06 | 1 | 0.81 | Yes (109; 100%) | 15 (13.76%) | 94 (86.24%) | 0.80 | 1 | 0.37 |
|  | No (166; 100%) | 128 (77.11%) | 38 (22.89%) |  |  |  | No (27; 100%) | 2 (7.41%) | 25 (92.59%) |  |  |  |
| **Psychosocial factors** | Yes (75; 100%) | 65 (86.67%) | 10 (13.33%) | 5.76 | 1 | 0.02 | Yes (41; 100%) | 5 (12.20%) | 36 (87.80%) | 0.005 | 1 | 0.05 |
|  | No (129; 100%) | 93 (72.09%) | 36 (27.91%) |  |  |  | No (95; 100%) | 12 (12.63%) | 83 (87.37%) |  |  |  |

Abreviations: n, number of participants; Chi^2^, value of the Chi2 statistic; df, degrees of freedom; p, test probability.
